# Food Loss and Waste Reduction in Specific Fruit and Vegetable Value Chains in Eastern Africa

**DOI:** 10.3390/foods14223938

**Published:** 2025-11-18

**Authors:** Willis Owino, Peter Kahenya, Elizabeth Wafula, Geoffrey Otieno

**Affiliations:** 1 Department of Food Science and Technology, School of Food and Nutritional Sciences, College of Agriculture and Natural Resources, Jomo Kenyatta University of Agriculture and Technology, P.O. Box 62000, Nairobi 00200, Kenya; 2 Department of Agriculture and Resource Economics, School of Agriculture and Environmental Sciences, College of Agriculture and Natural Resources, Jomo Kenyatta University of Agriculture and Technology, P.O. Box 62000, Nairobi 00200, Kenya; gotieno@jkuat.ac.ke

**Keywords:** food loss and waste (FLW), East Africa, tomato, mango, indigenous leafy green vegetables

## Abstract

The United Nations Sustainable Development Goal (SDG) 12.3 and the Malabo Declaration both address the critical issue of food loss and waste (FLW), but they differ in scope, timelines and regional focus. While SDG 12.3 provides a global framework and target of 2030, the Malabo Declaration reflects Africa’s pressing need to reduce FLW by 2025. Despite these targets and focus on FLW reduction by the global community, high FLW of fruits and vegetables continues to persist in many parts of Africa due to systemic constraints related to limitations in governance, financing and knowledge. It is estimated that up to 50% of nutritious fruits and vegetables are lost and yet supply hardly meets demand. The objective of this review was to identify the causes of FLW as well as possible solutions to reduce FLW in three fruit and vegetable values chains in East Africa. These three fruit and vegetable value chains were categorized as (i) “exotic, produced all year round, highly perishable and very inexpensive”, (ii) “exotic and indigenous, seasonal production, somewhat perishable and somewhat expensive” and (iii) “indigenous, produced all year round, extremely perishable and inexpensive”, represented by tomatoes, mangoes and indigenous leafy green vegetables, respectively. The upstream (farmer to market place) and downstream (market place to fork) causes of FLW are discussed and their respective solutions are suggested. The solutions provided herein are not only economically viable but also practical and can be adopted for the reduction of FLW in fruit and vegetable value chains in East Africa. This approach could result in the simultaneous increase of the access and affordability of fruits and vegetables for low-income consumers in East Africa.

## 1. Introduction

### 1.1. Importance of Food Loss and Waste

Food loss and waste (FLW) is also known as “food supply chain losses” to depict the different stages along the food chain at which a given proportion of food initially meant for consumption does not reach the intended consumer [1]. Furthermore, this work adopts previously differentiated definitions for FLW. Food loss happens in the upstream stages of the supply (farm, aggregation centers, during transportation and at wholesale and retail markets), while food waste occurs in the downstream stages, which involve the consumption level (restaurants and households) [2]. Food waste is a predominant phenomenon in high income countries, whereas food loss is mostly occurring in developing countries, caused by many factors [3]. These include and are not limited to highly fragmented small-holder production, poor handling practices, low levels of knowledge on proper handling, limited financing options to facilitate technology adoption, poor infrastructure, poor market forecasting brought about by misinformation by middlemen, low industrial processing capabilities and low exports, among others [4,5].

As a result, the quantity and caloric value of food lost in Sub-Saharan Africa (SSA) has been reported to be about 15.9% and 17.2%, respectively. Moreover, the percentage of FLW for fruits and vegetables is very high due to their delicate and perishable nature [5]. The occurrence of high FLW in fruit and vegetable value chains is a concern in a context of ongoing struggle to feed an ever-growing population. There are also rising dietary shifts characterized by a decrease in daily consumption of large quantities of starch-rich staples and an increase in the intake of micronutrient and fiber-rich fruits and vegetables, a phenomenon occasioned by economic development and globalization [6]. This implies that more fruits and vegetables will be demanded. High demand together with low supply are likely to push prices higher, further widening food access inequality gaps, which negatively impacts food security. In addition, FLW represents a waste of resources as the food supply chain is resource intensive and at the global level requires 20%, 70% and 32% of land, water and energy, respectively [7]. In addition, FLW negatively impacts the environment through increasing greenhouse gas emissions and pollution [8].

Therefore, it can be seen that FLW is a serious problem with multifaceted negative consequences which calls for concerted efforts to solve [9]. For this reason, among the 17 sustainable development goals (SDGs) established to achieve economic growth, social integration and environmental protection, SDG 12 focuses on ‘responsible production and consumption’. This SDG directly addresses FLW management in SDG 12.3. The goal of SDG 12.3 is to halve global per capita food waste at the retail and consumer levels by the year 2030 [10]. While SDG 12.3 sets a global benchmark, the Malabo Declaration reflects Africa’s urgent need to tackle FLW. One of the aims of the Malabo Declaration, adopted by the African Union (AU) in 2014, is to reduce FLW and halve post-harvest losses by 2025 [11]. Some progress has been made to meet these targets at the policy and strategic development level. For instance, a number of African nations have incorporated food loss and waste reduction objectives into their National Agricultural Investment Plans (NAIPs) under the Comprehensive Africa Agriculture Development Programme (CAADP). Furthermore, the African Union Development Agency (AUDA-NEPAD) and the Food and Agriculture Organization (FAO) support initiatives such as the Save Food Africa Programme in reducing post-harvest losses. While some countries have made strides, the continent as a whole is unlikely to meet the 2025 target of halving FLW due to systemic challenges, including (i) slow implementation progress due to funding and weak political enforcement, (ii) there currently being no standardized way of measuring food loss and waste at the country level, (iii) smallholder farmers having limited access to technologies and markets and (iv) the possibility of climate change and infrastructure deficits exacerbating the FLW [12]. In principle, FLW reduction would improve food security due to the increased availability food at reduced prices. As a result, the reduction, rescue or redistribution of food waste has the potential to increase access to food while improving nutrition and diet quality [6].

### 1.2. Scope of the Review

Efforts to reduce FLW have to start by first establishing the causes and the solutions aimed at their reduction. The causes for FLW are numerous and depend on the specific food value chain and region or country. This study is inspired by the case of Kenya, where 30–40% of FLW in the fruit and vegetable value chains has been reported. TechnoServe Inc. screened 40 of the top produced fruits and vegetables in Kenya and prioritized value chains based on origin, i.e., indigenous or exotic, seasonality, perishability and the level of inexpensiveness. In addition to the indicated criteria, the value chains were selected based on the potential for export and also on a high share of female participation. The three value chains selected were tomato, mango and indigenous leafy green vegetables (ILGs). Most of the indigenous leafy green vegetables (ILGs) are native to Africa, while others were introduced over a century ago and play a vital role in SSA nutrition and diet. The most common ILGs include Spider plant (*Cleome gynandra)*, Amaranth (*Amaranthus hybridus*), Cowpea leaves (*Vigna unguiculate*) and Nightshade (*Solanum scabrum*), among others [13].

Therefore, this review focuses on the FLW of the three value chains in Kenya (Table 1) by incorporating key lessons from Kenya and other East Africa countries.

### 1.3. Document Search and Screening Strategy

This review included articles from 2000 to 2025 as the timeframe during which the millennium development goals and sustainable development goals were adopted as well as the time during which discussions on food loss and waste were beginning to be widely initiated globally. This is because these goals and global discussions largely influence priorities for socio-economic and environmental policies, research and interventions. The studies selected on causes of food loss and waste in the three value chains of tomato, mango and ILGs included only those that looked into FLW in the East African region of SSA. This region includes Kenya, Uganda, Tanzania, Rwanda, Burundi, Ethiopia, South Sudan, Cameroon, Somalia, Eritrea and Djibouti. The studies on the solutions included not only those conducted in East Africa but also other parts of the world, as it was deemed that the solutions could appropriately address the causes of FLW in East Africa.

In addition, peer reviewed journal papers, conference proceedings, grey literature such as project reports and PhD and Master theses were included in this review. Opinion pieces, information on websites and news reports were excluded.

The search terms used were tomato, mango, African traditional vegetables, indigenous vegetables, food loss and waste and East Africa. The databases used to obtain articles included Scopus, Web of Science and Google Scholar.

A one-step screening of titles and abstracts was carried out by three researchers. This was followed by a full text screening of the studies. Then, the studies were read several times to understand meanings, develop themes and extract specific text segments/data, which were coded by three researchers while the fourth researcher crosschecked the coding. A data matrix was developed in which the themes were the columns and the data codes were added. This data matrix was analyzed using frequency counts prior to writing.

## 2. Tomato (*Solanum lycopersicum*)

### 2.1. Causes of Post-Harvest Losses and Waste of Tomato in East Africa

Table 2 describes the presentation of available data on the percentage post-harvest losses for mango and the causes in East African countries, based on the indicated research studies.

**Table 2:** Quantities of the post-harvest losses of tomato in different countries in East Africa.

Country	Study Description	Percentage of Post-Harvest Losses of Tomato	Indicated Causes	Reference
Kenya	Survey of sixty-eight farmers and traders	Sorting and grading: 22% Packing: 10% Transportation: 20% Decay: 20% Total loss: 72%	Decay due to fusarium rot and bacterial soft. Pests including fruit worms, spider mites, thrips, white fly, birds	[14]
Uganda	Key informant interviews including sixty participants from four districts of Uganda (Sheema, Nakasongola, Kabale, Luwero)	At harvest: 6% Transportation: 17% Market: 16%	Packing in wooden boxes, poor road access, ambient conditions	[15]
Ethiopia	FAO load tracking and sampling method involving key informant interviews, observation and load tracking. The study areas were Chimba, Gumara and Kudmi in Northwest Ethiopia	Farm: 6 to 9% Transportation: 1 to 8% Wholesale: 3 to 4% Retail: 9 to 13%	Handling tomato at ambient conditions of high temperature (23 to 25 °C) and low relative humidity (30 to 54%) among others	[16]
Ethiopia	Commodity systems analysis method, which involved a survey including one-to-one interviews including ninety-nine tomato producers (of which thirty were females), seventy traders and one hundred and twenty-nine consumers	Field: 10% per ha Total: 21% per ha	Market delays Climate fluctuations	[17]
Rwanda	Commodity systems analysis method including 10 farms and 10 markets in which 32 people (agronomists, farmers, wholesalers, retailers) were interviewed	Farm: 21% Collection points: 12% Whole sale markets: 10% Retail markets: 14% Total: 56%	Six causes were high lighted including: 1. Poor market integration leading to over-reliance on middlemen and agents 2. High cost of manufacturing and competition from cheaper products from neighboring countries: Kenya, Uganda and Italy. 3. Poor harvest practices including harvesting over-mature fruits, rough handling, overfilling harvest containers and multiple handling 4. Poor quality containers 5. Rough and long transportation with delays of 3–4 days before pick-ups and 3–9 h long drives 6. Lack of temperature control	[18]

#### 2.1.1. Pre-Harvest

Unavailability of certified seeds, open field production and failure to stake, which prevents tomato fruits from direct contact with soils, result in higher incidence of pests and diseases [16,19]. *Fusarium* rot and bacterial soft rot were cited by farmers as common diseases leading to post-harvest losses [14]. Additionally, low adoption of irrigation and greenhouses increased the weight loss of tomato [20]. Ochilo et al. [21] demonstrated that farmers in 121 major tomato growing areas in Kenya did not select varieties based on their shelf life stability yet variety has a significant effect on color development, firmness and disease incidence and/or severity post-harvest [22].

#### 2.1.2. At-Harvest

Farmers in Shewa, Ethiopia, harvested tomato throughout the day [17]. Furthermore, 22% of farmers surveyed in Kirinyaga, Kenya, harvested in the afternoon [14]. Harvesting when temperatures are high accelerates biochemical reaction rates leading to increased deterioration [23].

Different studies undertaken in Kenya [14] and Ethiopia [16,17] showed that farmers altogether harvested mature green, pink, ripe and over-ripe tomato fruits. The reasons for this were that mature green and pink tomato fruits had less incidence of disease and were easier to transport while ripe tomato achieved better prices from restaurants and hotels [24]. High ethylene production of the riper fruits hastens ripening of mature green fruits and in turn causes faster deterioration. For this reason, mixed harvesting of fruits is discouraged. Farmers in Tanzania delayed harvesting leading to over-ripe tomato fruits on farms. Cited contributing factors to delayed harvesting included inadequate access to costly solar powered storage facilities as well as socio-cultural concerns, i.e., low acceptability of frozen/chilled tomato [25].

Farmers and hired laborers had no prerequisite training on proper tomato harvesting leading to physical damage of fruits [17,18]. In one study, tomato fruits were thrown into harvest containers with the impact increasing damage [16]. In other studies, poor quality, unclean and/or rough harvest containers were used. These included woven reed baskets, wooden crates, buckets smeared with cow dung and jute or plastic bags [16,18].

#### 2.1.3. Post-Harvest

In Abera et al. [17], 38% of Ethiopian farmers surveyed were unaware of simple post-harvest treatments such as washing, which removes dirt and reduces the incidence of disease. Sorting is also an essential post-harvest step. Farmers in a survey carried out in Kenya separated healthy and diseased tomato fruits but no sorting according to the maturity stage was done [14].

Price misinformation caused by brokers and subsequent disagreements with farmers delayed the transportation of tomato fruits in Sidama, Ethiopia [26]. During such delays tomato fruits were stored at ambient temperatures, accelerating their deterioration [17,18]. Furthermore, packing in paper cartons with poor mechanical strength predisposed tomato fruits to bruises, compressions and vibrations [14]. It was also reported that packing tomato for transport was done in previously used wooden boxes that had been neither cleaned nor disinfected, putting the produce at risk of microbial contamination. Additionally, these boxes were overfilled and when stacked in vehicles approximately 2.18 kg and 3.44 kg were damaged in the middle and bottom stacked boxes, respectively, after transportation [17].

Different modes of transportation included carts and trucks, which were used 61% and 39% of the time, respectively, in Shewa, Ethiopia [17]. In Kenya, the most common means of transport was pickups, used 57% of the time, followed by lorries (29%), motorbikes (7%), carts (4%) and bicycles (1%) [14]. Rough handling and spillage during loading and offloading, poor road conditions, overloading and multiple handling (farmers to brokers, brokers to wholesaler and wholesalers to retailers) were identified as contributors to loss during transport [15,17,18,26,27].

#### 2.1.4. Market: Wholesale and Retail

Limited availability of cold storage facilities that can provide the recommended tomato storage temperature of 10 to 18 °C was noted [16]. High temperature, 22 to 25 °C, low relative humidity, 38% to 54%, and poor sanitation caused increased tomato deterioration on Ethiopian markets [16]. In addition, consumers were unwilling to buy poor quality tomato fruits, which were characterized as diseased, having an undesirable color, being deformed in shape and/or damaged during transport, leading to increased waste of tomatoes on markets [17,28]. Moreover, wholesalers and retailers in Ethiopia attributed the loss of tomato fruits to poor demand forecasting capabilities [26].

#### 2.1.5. Consumer Level

Eighty consumers in Sidama, Ethiopia, bought only one or two kgs of tomato for daily use at home. This implies that there might have been no reported waste, whereas café and hotels bought overripe tomato every two days and an estimated 1% to 5% was wasted [26].

### 2.2. Solutions of Post-Harvest Losses and Waste of Tomato in East Africa

#### 2.2.1. Pre-Harvest

To cope with the negative effects of climate change, farmers in Taita Taveta, Kenya, adopted the following mitigation strategies: early land preparation, adjusting of planting dates, planting early maturing tomato varieties, timely weeding and use of greenhouses, which can be limited due to the high cost implication [24]. In addition, grafted tomatoes demonstrate lower rates of weight loss, softening, color development and ethylene production than non-grafted ones [29].

Farmers using greenhouses produced 16.1 kg of tomato per m^2^ versus 2.3 kg per m^2^ produced by open field farmers [30]. Commercial farmers incurred 1% loss of tomato in comparison to 3% loss by subsistence farmers, partly attributed to greenhouse production and planting disease-resistant varieties [19]. Furthermore, a study carried out in Uganda, identified disease-resistant tomato genotypes. MT56 demonstrated resistance to bacterial wilt, whereas high yielding AVTO170 had resistance to *Talstonia solanacearum*, *Pseudomonas syringae* pv. and *Phytophthera infestanse* [31].

#### 2.2.2. At-Harvest Solutions

Ideally, tomato fruits should be harvested at the mature green stage as this leads to high retention of quality, freshness and marketability for long periods [32]. Mixed harvesting should be discouraged. Ripe tomato produces higher quantities of ethylene and may induce ethylene production in green tomato, accelerating ripening and subsequent deterioration [23]. In addition, harvesting in the morning into smaller containers of 20 kg, precooling within two hours after harvest and cold storage resulted in tomato fruits with an extended shelf life of 37 days and low post-harvest losses of 75 kg/ton [33].

Simple rinsing with clean water only has been reported to reduce many disease-causing agents of fruits and vegetables [23]. In addition, Orwa and Njue [34] showed that washing tomato with a concentration of 0.04 g of locally available Candle Brush per mL of water increased the shelf life from 9 to 14 days, significantly reduced the total viable count populations and delayed the development of off odors during the storage of tomato at ambient conditions.

#### 2.2.3. Post-Harvest Handling

Research has been dedicated towards evaluating low-cost approaches to cold storage such as evaporative cooling. Awafo et al. [35] reported that an evaporator cooler achieved temperatures and relative humidity of 23 to 27 °C and 81% to 96%, respectively, resulting in reduced weight loss of tomato fruits. The higher the cooling efficiency of the evaporative cooler, the higher the reduction in the weight loss of tomato [33,36]. In addition, evaporative cooling slows down the ripening and subsequent degradation of ascorbic acid, carotene, total phenolic and antioxidants contents [37], improving the post-harvest marketability of tomato [38]. Other cooling technologies tested in Ethiopia included the zero-energy cooling chamber (ZECC) and the pot in pot (PIP) refrigerator with the latter being cheaper and more effective at reducing ambient temperatures than the former [39].

The use of plastic crates to pack tomato should be promoted as they are easy to clean/disinfect, allow for air circulation and are smooth and thus reduce mechanical damage to the fruits, increasing the earnings of farmers and traders. An additional benefit in comparison with wooden crates is durability [40].

#### 2.2.4. Agro-Entrepreneurship

Inadequate market access can be overcome by promoting agro-entrepreneurship that combines breeding, production, aggregation and processing. Such an inclusive agri-business model consolidates production of 100 to 500 smallholder farmers and can overcome challenges of rural financing. Njoro Canning Limited, based in Loitoktok (Kenya), owns large tomato-producing farms and also aggregates produce from small scale farmers and transports them to their processing plant. Additionally, producers’ associations in Kirinyaga and Muranga, Kenya, have successfully aggregated produce for transport to processing facilities in Nairobi [41].

Dash Industries contracted tomato farmers to produce the Tanya variety, which is suitable for processing [42]. The company produces tomato pastes under the brand name Red Gold and sells the seeds to seed companies. This integration of a value chain allowed for exploitation of a new market segment in Arusha, Tanzania [43]. Furthermore, increasing farmers’ access to financing motivates tomato processing [24]. Industrial processing of tomato results in high retention of micro-nutrients such as magnesium, manganese, phosphorus, potassium and iron and higher total phenolics and flavonoids [44].

## 3. Mangoes (*Magnifera indica* L.)

### 3.1. Causes of Post-Harvest Losses and Waste of Mangoes in East Africa

#### 3.1.1. Pre-Harvest

Farmers inability to identify pests and diseases and therefore apply effective prevention and control measures has been reported to lead to increased losses of mango fruits in East African countries [45]. The most widely reported pest is the fruit fly (*Bactrocera dorsalis*). Fungal diseases such as white scale (*Aulacaspis tubercularis* Newstead), sooty mold and powdery mildew as well as anthracnose, a bacterial disease, and parasitic fungi have been cited as major causes of fruit loss [45].

Table 3 describes the presentation of available data on the percentage post-harvest losses for mango and the causes in East African countries, based on the indicated research studies.

**Table 3:** Quantities of the post-harvest losses of mangoes in East Africa.

Country	Study Description	Percentage of Post-Harvest Losses of Mangoes	Indicated Causes	Reference
Ethiopia	Assessment of mango post-harvest losses along value chain in the Gamo Zone, Southern Ethiopia	Disease and pests:7% Harvesting: 18% Collection: 9% Transportation: 7%	Daily laborers harvest both mature and immature mango via mixing. Traditional ways of harvesting mechanisms like shaking of the mango tree, picking with a stick and cutting the fruit branch. Use of traditional ways of transport.	[45]
Ethiopia	The study was conducted in four mango-producing districts selected from two geographic regions, viz, Eastern and Western Ethiopia. Accordingly, four districts that encompassed 13 villages were purposively selected. A total of 113 mango grower households that represented 15% of the identified potential mango growers of each district were randomly selected	Pest and diseases: 66% Poor transportation:5% Limited knowledge and skills: 50.4%	Most growers harvest fully ripe fruits. Poor harvest and handling practices could result in various blemishes on the fruit skin. Use of traditional ways of transport.	[46]
Tanzania	Evaluation of post-harvest losses and shelf life of fresh mango (*Mangifera indica* L.) in the eastern zone of Tanzania. Fruits were harvested from Lunyala village in Mwalusembe ward (7.1435° S, 39.0542° E and 58.30 m above sea level) in Mkuranga district, coast region in eastern Tanzania. Apple and Palmer, the commonly grown cultivars in the study area, were used.	Loss at harvest stage: 3% (Apple) and 3% (Palmer) Loss at transport stage: 9% (Apple) and 9% (Palmer), Wholesale stage: 8% (Apple) and 8% (Palmer) Retail stage: 25% (Apple) and 23.01% (Palmer).	Crashing, bruising, infestation by insects such as fruit flies, over-ripening, post-harvest diseases and scratching by farm tools.	[47]
Kenya	Assessment of potential and limitation of post-harvest value addition of mango fruits in eastern province: a case study in Embu and Mbeere districts.	Total loss: 45% after harvest	Limited handling skills, poor storage, pests and diseases, e.g., 49.4% of farmers determined maturity of mango fruits by hand feeling, while 41.6% determined by size of the fruit.	[48]

#### 3.1.2. At-Harvest

Growers in Kenya and Ethiopia were mostly unaware of mango maturity indices [46,48]. In Kenya, 49% of farmers determined maturity subjectively by hand, 42% measured fruit size and only 5% considered mango shoulders, an accurate indicator of mango maturity [48]. Furthermore, Kassa and Fasika [45] revealed that during harvesting, laborers in Goma, Ethiopia, mixed mature and immature mangoes. Harvesting immature mango prevents the fruit from reaching full maturity, while harvesting over-ripe mango shortens the shelf life due to the susceptibility to bruising and decay [49].

Hand harvesting was reported to be a common practice [46]. Krishnapillai et al. [50] observed that manually severing the mango fruit from the stem causes the release of resin for up to one hour. The sap, which has a low pH and is thus acidic, burns the fruit surface causing the occurrence of red and black spots and peel decay, compromising the fruit’s acceptability on the market.

In Ethiopia, harvested mango fruits were mainly stored under mango trees (58.4%) or storehouses (41.6%) constructed from local materials at ambient conditions conducive for deterioration [46].

#### 3.1.3. Inappropriate Post-Harvest Handling

In a study carried out in Ethiopia, synthetic fiber sacks were the most common packaging material (80%) used and resulted in bruising of the fruits [46]. Inappropriate packaging materials may cause mechanical damage which manifests as spongy tissue which becomes visible when the mango fruit is cut open [51].

Sivakumar et al. [52] demonstrated that improper packaging, transport and inadequate field handling practices significantly contributed to post-harvest losses and poor organoleptic, nutritional and techno-functional characteristics of mango fruits. In Tanzania, high post-harvest losses of the Apple mango cultivar occurred during the transport stage because of crashing, bruising and scratching caused earlier on by farm tools [47].

#### 3.1.4. Waste of Mango at Consumption and Value Addition

Processors rely on brokers and in some cases organized farmer groups for delivery of mango. Upon delivery to the factories, mangoes are weighed, inspected and sorted according to the processing requirements, specifically sugar content. Losses at this stage are estimated at 10–31% in Kenya with higher losses observed for farmer deliveries in comparison to those for brokers who carry out pre-selection of mango fruits [53]. Common rejection reasons include immature fruit, insect/pest damage, over-ripeness and bruising [54]. Furthermore, Kenya’s equipment manufacturing industry is limited, and as a result, dryers, freezers, pasteurizers and sterilizers must be imported. This hampers the development of the mango processing infrastructure, thus leading to mango losses especially during periods of glut [53]. The processors face strong competition from fresh and export markets, where higher prices can be paid [55].

### 3.2. Solutions of Post-Harvest Losses and Waste of Mangoes in East Africa

#### 3.2.1. Pre-Harvest

Pruning decreases the disease incidence of both anthracnose and stem-end rot in ripe fruits stored at room temperature [56]. Understanding pest and disease aspects of horticultural crops is essential for designing effective integrated pest control strategies. Integrated pest management technology has been developed by the International Centre of Insect Physiology and Ecology (ICIPE) and partners to suppress mango fruit flies on smallholder mango farms in SSA [57]. Application of Mancozeb and Prochloraz at the pre-harvest stage is considered an effective measure to reduce anthracnose disease of mango fruit [58]. Pre-harvest bagging of mango fruit is primarily done for physical protection from fruit fly, but it also influences fruit quality to some extent by promoting peel color and reducing skin blemishes through changing the micro-environment of fruit [59].

Mango fruit should be harvested when mature, but not ripe, based on the fullness of the cheeks, skin glossiness, flesh color, development of the shoulders and number of days from bloom to harvest [60,61]. Moreover, green maturity indices should be developed for each specific mango variety [52].

#### 3.2.2. At Harvest

Picking fruits with stems slightly above the abscission point is recommended. The fruits in different parts of the tree should be harvested one by one with a bamboo-pole harvester and collected in a net-bag attached to it. Harvesting in the morning is the best time to minimize the sap burn injury to the mango skin [62]. Gentle harvesting of mangoes should be carried out using picking poles with bags attached to prevent sap burn and mechanical damage that may lead to increased incidence of deterioration and hence losses [49,63].

#### 3.2.3. Post-Harvest Handling

Systematic sorting and grading, combined with appropriate packaging and storage, contribute to increased shelf life and maintenance of quality and associated marketing costs [64]. Sorting serves the purpose of classification based on various quality factors, such as size. With changing consumer preferences and the need to reduce losses, proper post-harvest handling is essential [60]. The growth of supermarkets and institutional buyers emphasizes the importance of post-harvest handling to meet quality demands [49].

Post-harvest pretreatments can be either non-chemical and chemical in nature [49]. Dipping the mango fruits in hot water at 50–53 °C for 15 min is an effective post-harvest treatment [60].

The study of Orjuela-Castro et al. [65] emphasized the important role packaging plays in the ripening of mangoes. One study demonstrated that cartons lined with polythene bags resulted in the lowest weight loss in mangoes stored for six days at 31 °C compared to polythene bags and unpacked mangoes. Packaging with polythene bags only still resulted in lower weight loss than no packaging at all [66].

Cold storage at a temperature range of 10–13 °C, depending on the mango cultivar, could extend the shelf life for 2–3 weeks [67]. Evaporative cooling can also be used for mangoes [68] as is the case for tomato fruits. An evaporative cooling technique named Coolbot™ has been considered an effective approach for preserving Apple and Ngowe mango cultivars, extending the shelf life to 35 days compared to fruits stored in ambient room conditions [69]. Being part of a cooperative can increase the affordable accessibility of evaporative coolers as demonstrated by small-holder farmers in Kenya, which resulted in increased awareness and use of evaporative coolers. It should be noted that the loss reduction rate and/or income increase was not documented in this study [70]. Controlled atmosphere storage (CAS) has also been applied to prevent chilling injury in mangoes [71].

#### 3.2.4. Value Addition of Mangoes

Post-harvest losses of mango can be further mitigated through value addition. This includes practices such as adopting simple processing techniques such as drying and pulping, offering subsidized loans to exporters, reducing tariffs on imported equipment and training farmers, traders and processors on phytosanitary requirements to increase marketability. Moreover, Kenya contributes 2% of global mango production and 1% of mango exports. Growing the mango export market to include value added differentiated products such as dried mango and mango pulp could expand the export sector [53].

## 4. Indigenous Leafy Green Vegetables (ILGs)

### 4.1. Causes of Post-Harvest Losses and Waste of ILGs in East Africa

#### 4.1.1. Pre-Harvest

How breeding improved varieties of ILGs has received little attention [72]. Farmers in Nakuru, Kisii and Kakamega, Kenya, cited a lack of improved varieties as a cause of post-harvest loss. Poor seed quality causes farmers to continue to rely on retained seeds of landraces due to institutional seed suppliers’ delayed entry into the market [73]. In one study, the unavailability of good quality seeds for preferred Amaranth varieties in Kenya and Tanzania has been reported [74]. With the limited availability of better cultivars and the continuous usage of landraces, productivity (yield per unit land) remains low [75]. In addition, unfavorable weather conditions and a high prevalence of insects, pests and diseases also cause post-harvest losses [76].

Table 4 describes the presentation of available data on the percentage post-harvest losses for Indigenous leafy vegetables and the causes in East African countries, based on the indicated research studies.

**Table 4:** Summary of the causes of post-harvest losses of ILGs in East Africa.

Country	Study Description	Type of FLW	Indicated Causes	Reference
Kenya	Assessed the relative potential for formal or informal seed systems to meet the need for high-quality indigenous vegetable seed	Pre-harvest	Seed quality of available formal and informal seed varieties	[73]
Kenya	How value chain governance influences farmer participation in vegetable markets and food security	Pre- and post-harvest	Value chain governance	[77]
Kenya	Effects of commercializing African indigenous vegetables in Kenya	Post-harvest	Education, participation in producer groups, access to market information and irrigation water, as well as distance to the next city influence the decision to commercialize ILG production	[78]
Kenya	Understanding consumer acceptance of ILGs is important in enhancing their consumption	Post-harvest	Consumer acceptance of leafy African indigenous vegetables	[79]
Tanzania	Primary survey of growers in Tanzania	Pre-harvest	Nutritional perception and marketing	[80]
Kenya	Quantitative data analysis of African indigenous vegetable (ILG) farmers		Adaptive capacity of smallholder African indigenous vegetable farmers	[81]
Tanzania	Descriptive statistics to measure awareness and perception of solar-dried vegetables	Post-harvest	Decisions to purchase solar-dried traditional African vegetables	[82]
Kenya	Evaluation of the major pests attacking leaf amaranth and African nightshades and their potential host ranges.	Post-harvest	Yield losses due to viral, bacterial, fungal diseases and arthropod	[83,84]
Kenya	Determined the effectiveness of evaporative cooling using zero energy brick cooler (ZEBC) and evaporative charcoal cooler (ECC), to preserve the post-harvest quality of leafy amaranth vegetables	Post-harvest	Preservation of post-harvest quality	[13]
Eastern Africa	A qualitative literature review of indigenous vegetable seed system	Pre-harvest	Seed systems of African vegetables	[85]
Sub-Saharan Africa	Improvement and development strategy for traditional African vegetables in sub-Saharan Africa	Pre-harvest	Breeding	[86] et al. (2016)
Kenya	Post-harvest losses attributed to inadequate conditions during transport, storage and marketing	Post-harvest	Post-harvest treatments and handling	[76,87]
Kenya	Role of indigenous vegetables in food and nutritional security	Pre- and Post-harvest	Food degradation, monotonous food supply and emergence of vitamin and mineral deficiencies (hidden hunger).	[88]
Uganda and Kenya	Narrative literature review approach on current trends in post-harvest management and processing	Post-harvest	Post-harvest handling and processing	[89]
Tanzania	The role of community-based nutritional outreach	Pre-harvest	Changing knowledge and perceptions of African indigenous vegetables	[90]
Kenya	Physiological status, storage temperature and duration affect phytonutrient levels and post-harvest life of the leafy vegetable.	Post-harvest	Development stage, storage temperature and storage duration affect phytonutrient content in cowpea	[72]

#### 4.1.2. At Harvest

Mechanical damage during harvest creates entry points for pests and raises physiological losses. In a study carried out in Uganda, bruises and injuries during harvest were reported to be a major cause of on-farm post-harvest loss of ILGs [91].

Findings of a survey indicated that the farmers who harvested ILGs in the afternoon when temperatures are high and did not keep them in the shade experienced very high post-harvest losses [76]. The aforesaid study also reported that the majority of farmers harvested ILGs early morning, which is also ill advised for this vegetable crop due to the presence of dew which may later cause a proliferation of microorganisms causing diseases [76].

#### 4.1.3. Inappropriate Post-Harvest Handling

It was reported that African nightshade was washed and drained before being transported to the market. This practice resulted in 0 to 3% post-harvest loss of the vegetables. Furthermore, these vegetables were packed in large white sacks of poor mechanical strength and 6.4% of the ILGs were lost in transit due to physical damage [92]. Most farmers in Kenya used conventional non-perforated polyethylene bags and gunny bags to package ILGs, leading to heat build-up and deterioration of produce [76].

Farmers were reported to be aware of the need for cold storage; however, they did not utilize cold storage facilities because of high up-front costs. Furthermore, most farmers were unaware of the appropriate cold storage temperature for the specific ILGs they were handling [76].

Poor infrastructure, particularly road networks, has been identified as a major barrier in the transportation of ILGs [93,94]. Poor road conditions contribute to increased transit distance making timely market supply problematic and expensive [95]. Dropping of ILGs during transport to the market was noted as another cause of post-harvest losses [91].

#### 4.1.4. Market Factors: Wholesale and Retail

There are problems resulting from the supermarket “revolution” [96]. Supermarkets do not give technical or financial support to farmers who have little capacity to control vegetable quality [77]. Critical challenges also for ILGs include a shortage of defined market space and limited market knowledge on the quantity and pricing at any given moment [97]. One study highlighted the challenges to ILG product market penetration in certain areas [80] due to limited product differentiation, which can make them more appealing to customers.

Market hygiene is another major concern. Unsanitary conditions create a conducive environment for the growth and multiplication of disease-causing agents such as bacteria soft rot (*Erwinia* spp.) and grey mold (*Botrytis cinerea*) in addition to infestation of pests including aphids, white flies, flea beetles and red spider mites [76].

Market potential, consumer attitudes and willingness to pay are critical factors [98]. Changes on the consumer side, such as urbanization and the growth of the middle class, impact demand for indigenous foods [88], as consumer willingness to buy ILGs decreases with higher income levels [99].

#### 4.1.5. Consumption Level

Post-harvest losses of African nightshade were reported to be highest at the point of consumption due to the removal of edible stems and fruits, resulting in a 25% physical loss. The most commonly adopted method of preparation was boiling for 30 to 60 min followed by frying, likely resulting in high vitamin C losses [92].

#### 4.1.6. Gender-Related Factors

A study in Kenya put forward that though women dominate the ILG value chain, they tend to face many challenges which contribute to the loss and waste of the vegetables. Women are less likely to own farms and face discrimination, insecurity and/or displacement by men during production, transportation and marketing of ILGs as markets become lucrative. This limits the extent to which they can put in place measures to reduce loss and waste of ILGs [100]. This implies that solutions to FLW in ILGS value chains must be gender-sensitive to increase the solutions’ effectiveness.

### 4.2. Solutions of Post-Harvest Losses and Waste of ILGs in East Africa

#### 4.2.1. Pre-Harvest

Contrary to farmers reporting the unavailability of high-quality seeds on the markets, there are seed firms that are already producing certain improved ILGs. These include seeds for amaranth, African nightshade, jute mallow, Ethiopian mustard, cowpea leaf and spider plant [85,86]. Bringing novel new seed types to market needs commitment and institutional backing, such as support provided by the World Vegetable Center. To enhance the seed quality capacity, building towards improved breeding skills is needed as well as improved regulation and access to seed [101]. Seed regulation includes carrying out testing, certification, development, release and protection of varieties suitable for different agroecological zones and registration of vendors. This can be done through identification of gaps in different seed systems, i.e., private and community-based, and stakeholder engagement, in addition to policy review and development of an implementation framework that includes an enforcement strategy [83]. An increase in trust and dependence on sustainable private seed systems needs to be fostered [85].

In addition, identifying the individual pest species that target specific ILGs and differ by locality is a first step toward designing long-term integrated pest control techniques [84]. In addition to chemical pest control, locally made seed coatings, entomopathogenic fungi and biopesticides derived from plant products such as neem can be explored [81,102]. Farmers must also adjust their farm-level management to include aspects of climate change during the production of ILGs which are known to be relatively resistant to weather impacts [103].

#### 4.2.2. At Harvest and Post-Harvest Handling

Choosing the optimal maturity indices for each ILG plant species provides for increased post-harvest quality of the vegetables [88].

Systematic sorting or grading along with proper packing and storage will increase the shelf life, preserve the wholesomeness, freshness and quality and significantly minimize losses and marketing expenses [64].

In addition to functioning as a handling unit, packaging should be designed to minimize early deterioration of the product quality [87]. For packing ILGs, clean, smooth and well-ventilated containers should be used [64]. Modified atmosphere packaging (MAP) bags, when combined with biodegradable film packaging (PLA) bags, can minimize FLW in ILG value chains while maintaining the sensory and nutritional quality of the vegetables. At temperatures in the range of 0–5 °C, ILGs have the longest shelf life [76]. Farmers should employ simple and affordable cooling facilities, such as evaporative cooling with zero-energy brick coolers or evaporative charcoal coolers [13]. UV-C treatment of perishable ILGs is a novel method for extending the shelf life and quality of fresh horticulture crops [104].

#### 4.2.3. Marketing

It is critical to highlight that in ILG value chains, the durations for demand and supply information sharing may vary across geographical regions [78]. Proper and region-specific planning and collective action in coordinating and governing is required to accomplish efficient ILG transport coordination. Additionally, efficient handling of ILGs during transit necessitates knowledge of the acceptable circumstances as well as abilities for preserving the quality of ILGs. This will lead to the reduction of transport costs and increased knowledge sharing, resulting in benefit optimization among chain participants [105].

#### 4.2.4. Value Addition

Market differentiation and niche marketing of high-value ILGs are critical to the success and expansion of ILG consumption [80]. ILGs can be positioned for high-value specialized market niches [106].

## 5. Conclusions

This review has demonstrated that there are commonalities in the causal factors of post-harvest losses and waste of fruits and vegetables in East Africa. At pre-harvest, there is limited availability of good quality seed for preferred varieties, poor agronomic practices, inadequate funding to carry out protected agriculture and a high prevalence of pests and diseases. At harvest, many of the farmers and their laborers are not adequately conversant on appropriate harvesting techniques for specific commodities and there is rough handling of produce combined with inappropriate packaging, which predisposes the fresh fruits and vegetables to physical damage. Additionally, harvesting is delayed and is not carried out for fruits at the required maturity stage, mixed harvesting and packing is undertaken and simple post-harvest treatments such as washing are not practiced and produce is harvested directly for the market. Cold storage is simply out of the reach of many farmers because solutions are not economical and require high up-front costs. During post-harvest handling, the overfilling of crates and limited availability of transport on rough roads cause spillage and further bruising and scratches on the fresh produce. At the market, the inadequate availability of cold storage persists and poor sanitation leads to a proliferation of disease-causing agents and pests that cause losses of tomatoes, mangoes and ILGs. There are also limited processing or value addition options for farmers who are hesitant to take on these new avenues as the local acceptability of fresh fruits and vegetables is well established whereas there is uncertainty of the acceptability of processed or cold stored/frozen foods. In addition, consumer rejection of deformed produce or spoilt produce is yet another cause of loss and waste. Information on the causes of loss and waste at the consumer end is quite sporadic and further investigations of this are required in the future.

Based on this literature review we find that:

(1) Several causes of FLW and the corresponding solutions are common across all three fruit and vegetable value chains reviewed in this paper.
(2) More research is needed on the economic, social-cultural and policy solutions that contribute to reduction in FLW. These would comprise solutions that do not contribute to rebound effects (i.e., second-order market interactions, e.g., reduction in food prices) pushing FLW back up elsewhere in value chains nor weakening the incentives of the farmers to produce more food.

It is envisioned that this review will act as a starting point to identify and prioritize economically viable and practical solutions that reduce FLW. This could involve both existing and underutilized as well as potentially novel preventative and rescue solutions to increase the access to and affordability of fruits and vegetables for low-income consumers in East Africa. Preventive solutions are those solutions that stop food loss before it occurs by reducing inefficiencies and overcoming challenges along the value chain. The type of prevention depends on the activities in each commodity value chain stage. Rescue solutions are those that derive value from fruits and vegetables that would otherwise be thrown away. The type of rescue depends on the state of the produce when it is collected at harvest.

## Figures and Tables

**Table 1:** Rationale for value chain inclusion.

Value Chain	Rationale for Selection	Losses on Fresh Weight Basis Within Value Chains in Kenya
Tomato	Is the 3rd ranked vegetable in Kenya by production volume. Good sources of fiber, beta carotene, potassium and the antioxidant lycopene.	32% of tomato production volumes are lost along the value chain, representing approximately 309,000 tons of losses annually.
Mango	Ranks 3rd among fruits in Kenya by production. Good source of folate, beta carotene and vitamin C.	35% of mangoes is lost along the supply chain, representing a total volume of 283, 000 tons annually.
Indigenous Leafy Green Vegetables	Good source of micronutrients (e.g., iron, zinc, folate and provitamin A). Growing demand from awareness of nutritional benefits. High income opportunity for farmers and women.	34% of production volumes is lost along the supply chain, representing approximately 101,000 tons annually.

Source: TechnoServe Inc.

## Data Availability

No new data were created or analyzed in this study. Data sharing is not applicable to this article.
